# Differential networks for processing structural dependencies in human language: linguistic capacity vs. memory-based ordering

**DOI:** 10.3389/fpsyg.2023.1153871

**Published:** 2023-07-19

**Authors:** Keita Umejima, Isso Nakamura, Naoki Fukui, Mihoko Zushi, Hiroki Narita, Kuniyoshi L. Sakai

**Affiliations:** ^1^Department of Basic Science, Graduate School of Arts and Sciences, The University of Tokyo, Tokyo, Japan; ^2^Graduate School of Humanities and Sociology, The University of Tokyo, Tokyo, Japan; ^3^Japan Society for the Promotion of Science, Tokyo, Japan; ^4^Graduate School of Languages and Linguistics, Sophia University, Tokyo, Japan; ^5^Faculty of Cross-Cultural and Japanese Studies, Kanagawa University, Kanagawa, Japan; ^6^Department of English, Faculty of Letters, Tokai University, Kanagawa, Japan

**Keywords:** syntax, Merge, ordering, lateral premotor cortex, inferior frontal gyrus, fusiform gyrus, fMRI

## Abstract

Surface linear (left-to-right) arrangements of human languages are actually an amalgam of the core language system and systems that are not inherently related to language. It has been widely recognized that an unbounded array of hierarchically structured linguistic expressions is generated by the simplest combinatorial operation “Merge,” and the notion of Merge-generability has been proposed as a key feature that characterizes structural dependencies among linguistic elements. Here we tested Merge-generable dependencies by using a Subject-Predicate matching task, which required both linguistic capacity and short-term memory. We used three types of dependency: Nesting, Crossing, and Grouping as the control. The Nesting dependency is *totally* Merge-generable, while the Crossing dependency requires some additional processes for memory-based ordering. In order to identify the regions employed for these two dependencies, we directly compared cortical responses to the sentence stimuli (with noun phrases and an adverb as the first half of stimuli, and with verbs as the latter) using functional magnetic resonance imaging (fMRI), and the following results were obtained. First, for the Nesting – Crossing contrast, significant activations were observed in the bilateral lateral premotor cortices (LPMCs) and inferior frontal gyri, left middle temporal gyrus, and bilateral angular/supramarginal gyri, indicating engagement of the syntax-related networks. In contrast, the Crossing – Nesting contrast showed focal activations in the left fusiform gyrus, lingual gyrus, and middle occipital gyrus (L. FG/LG/MOG). Secondly, for the first half of the Nesting stimuli, signal changes in the bilateral LPMCs were well fitted with the estimates of computational costs to search the workspace and to select items (Σ operations). Moreover, for the latter half of the Crossing stimuli, the signal changes in the L. FG/LG/MOG were differentially fitted with the estimates of loads related to the ordering of elements/words (numbers of Ordering). Thirdly, these fitting models were by far more likely than the exchanged estimates between bilateral LPMCs and L. FG/LG/MOG, confirming a double dissociation for primary processes with Σ and Ordering. In conclusion, these results indicate that separate cortical networks are differentially employed, and their careful elucidation will provide further insights and challenges.

## 1. Introduction

The most significant feature of natural language syntax is the existence of abstract hierarchical structure. Linguistic expressions have abstract hierarchical structures, and those structures do matter. The human language faculty systematically makes use of hierarchical structure instead of, say, adjacency (being “closest” in terms of linear order), which seems to be a more easily accessible relation. Consider the following elementary phenomenon in English.

(1) a. The bombing of the cities *was* criminal.
  b. The bombings of the city *were* criminal. (Chomsky, 2021, p. 9)

If syntax employed adjacency for calculating subject-verb agreement, the pattern (2) below would be observed, where verbs agree with the adjacent noun (*city/cities*). However, all these examples are ungrammatical (an asterisk (*) indicates an ill-formed expression).

(2) a. *The bombing of the cities *were* criminal.
  b. *The bombings of the city *was* criminal.
(3) [[The bombing(s) [of [the cities/city]]] [was/were criminal]]

As can be seen from the structure (3), *city/cities* are deeply embedded in the subject noun phrase (NP), and thus they are hierarchically more “distant” from the verbs, compared to the NP headed by *bombing(s)* (i.e., *{the bombing(s) {of {the cities/city}}}*), even though *city*/*cities* are linearly closest (adjacent) to the verbs. The critical importance of abstract hierarchical structures, as opposed to left-to-right linear order, has been explicitly noted ever since the inception of transformational grammar (Chomsky, 1955). The structure-dependence property of linguistic rules, compared with artificial rules based on linear order, was confirmed by a functional magnetic resonance imaging (fMRI) experiment (Musso et al., 2003).

In current syntactic theory, it is widely assumed that hierarchical structures of human language are built by the operation *Merge*, which takes *n* (usually two) elements and forms an unordered set of them:

(4) Merge (X_1_, X_2_, …, X_n_) = {X_1_, X_2_, …, X_n_}.

We refer the reader to any introductory textbook on contemporary syntactic theory (Hornstein et al., 2005) for expositions as to exactly how an unbounded array of hierarchical structures is generated by this simple operation.

The simple facts in (1) and (2) above clearly demonstrate that dependencies in human language (in this case, the subject-verb agreement) are subject to the “minimality condition,” where the relevant “minimality” is defined over abstract structures [such as the one depicted in (3)] generated by Merge, rather than the minimality/adjacency defined in terms of surface linear strings. Linguistic expressions/sentences in human language are generated by Merge, and legitimate dependencies can be obtained only to the extent that they are characterizable over Merge-generated structures (Merge-generability). Thus, we can naturally make the following proposition, which expresses a “necessary” condition for any natural dependency in human language.

(5) The Merge-generability Hypothesis (Tanaka et al., 2019):
  Only Merge-generable dependencies are naturally computable as linguistic dependencies by the human language faculty.

The Merge-generability Hypothesis (5) states that all syntactic dependencies including agreement are determined with crucial reference to hierarchical structures generated by Merge (along with the general condition on minimality defined over structures, as we saw above). These types of dependencies are called “Merge-generable” dependencies (Tanaka et al., 2019).

On the other hand, dependencies that are not based on structures generated by Merge (for example, those dependencies solely based on linear order, ignoring hierarchical structures) are not processed naturally as linguistic dependencies, but are treated as a kind of puzzle/game (even though using legitimate lexical items). Note that the Merge-generability Hypothesis just mentioned predicts that certain particular regions are activated only when processing Merge-generable dependencies. Tanaka et al. (2019) conducted an fMRI experiment to test the prediction, and revealed different activation patterns when processing Merge-generable and non-Merge-generable dependencies, though they have left open exactly which region actually enables the formation of “partially Merge-generable” dependencies such as Crossing, which are based on structures generated by Merge but cannot be obtained by Merge alone. It is the main focus of the present study to specify the region that is responsible for the additional processing operation involved in Crossing.

This study experimentally shows that constructions exhibiting dependencies based on linear order (Crossing dependency) actually result from the application of the peripheral order-assigning operation called FormSequence (FSQ; see below for more details) to hierarchical structures built by the core syntactic operation Merge, and it also reveals the specific brain regions for FSQ-processing. The results provide further support for the Merge-generability Hypothesis proposed by Tanaka et al. (2019), according to which only Merge-generable dependencies are naturally available in human language. As briefly discussed above, our results also significantly clarify the nature of the distinction (made in Tanaka et al., 2019) between “totally Merge-generable” dependencies (e.g., Nesting) and “partially Merge-generable” dependencies (e.g., Crossing) by elucidating exactly what formal mechanism, in addition to Merge, is involved in the latter, explaining at the same time why the latter type of constructions are so rare and exceptional in human language.

### 1.1. Linguistic background

Let us here introduce and briefly explain the minimal theoretical machinery assumed in linguistics that we use below for analysis of the dependencies under consideration. The subject-verb agreement relation we discussed above is one notable dependency relation. Another typical dependency relation in human language is the Subject-Predicate relation. While involving similar elements, these two relations are different in nature. The former (subject-verb agreement) relation is a formal relation with possible morphological reflexes, whereas the latter—Subject-Predicate—relation determines which NP (indicated by capitalized “Subject”) semantically corresponds to which Predicate/V, forming a Subject-Predicate pair that leads to a specific semantic interpretation. In (1a) above, for instance, the Subject of the Predicate *be* is the NP headed by *bombing* (*the bombing of the cities*), not the one headed by *cities* (*the cities*). That is, just as in the case of subject-verb agreement, the structurally closest element—not the linearly closest one—is chosen as the “head” of the Subject. This general pattern can be uniformly captured by hypothesizing a single mechanism Σ (Search), applying to structures generated by Merge, and which is responsible for both subject-verb agreement and the formation of Subject-Predicate (S-P) pairs. Σ is a general—perhaps not specific to the language faculty—search mechanism, which, in this case, operates on structures provided by Merge. Σ applies in the following two-step fashion, in compliance with the very general minimality condition (Minimal Search), which states that “Σ searches as far as the first element it reaches and no further” (Chomsky, 2021, p. 18). We assume that Σ starts searching from the given S node, and that, if more than one S is involved, Σ applies cyclically.

(6) Σ (Search):
  a. Σ_1_: Search the closest X and identify it as the relevant element.
  b. Σ_2_: With X fixed, search Y in the smallest set containing X (its “sister” domain).

If Σ applies to the structure (3) to form an S-P pair, it starts searching from the root set *{{The bombing(s) {of {the cities/city}}} {was/were criminal}}*, and picks the highest NP *{the bombing(s) {of {the cities/city}}}* as the relevant element [*X* in (6a)]; with the Subject fixed, Σ then searches a Predicate in the sister domain of the Subject, i.e., *{was/were criminal}*, finds *was/were* [*Y* in (6b)], and finally forms a pair <*{the bombing(s) {of {the cities/city}}}*, *was/were*>. For the S-P pair-formation, we assume the operation Form Subject-Predicate (FS-P) as a special case of the general interpretive rule Relate based on Σ (which assigns a relation R to the pair <X, Y>, i.e., R<X, Y>). Here, the relation in question is “Subject-Predicate (S-P) pair.” See the Discussion section below. See also Chomsky (2021) for much relevant discussion.

(7) Form Subject-Predicate (FS-P): Assign the relation S-P to <X, Y> determined by Σ (6) (i.e., Y is the Predicate of the Subject X).

FS-P determines *was/were* as the Predicate of *{the bombing(s) {of {the cities/city}}}*. Note that these are very general and core properties of human language, and their accounts are provided by the fundamental (though very simple) operation Merge and the general Minimal Search mechanism (i.e., Σ working under the minimality condition).

The situation is a little bit different with the so-called Crossing dependency:

(8) [[*Taro-toKenta-ga*]*sorezore*[*sakebihanasiteiru*]]
  *Taro and Kenta-* NOM *respectively shouting uttering*
  “*Taro and Kenta are respectively shouting and uttering* (= *Taro is shouting, and Kenta is uttering*)”

In (8), the adverb *sorezore* “respectively” forces Crossing-dependency, effectively preventing Σ from forming the S-P pair <*{Taro-to Kenta-ga}*, *{sakebi hanasiteiru}*>. If FS-P were to apply, it could neither determine which NP (*Taro* or *Kenta*) would be X nor which V/Predicate (*sakebi* or *hanasiteiru*) would be Y. In order to solve this problem, Chomsky (2021, p. 31) proposes an operation FormSequence (FSQ). Unlike Merge, which is a fundamental structure-building operation, FSQ is required by a specific type of construction, and its applicability seems to be heavily restricted accordingly. FSQ assigns an ordering relation to the members of a set formed by Merge:

(9) FSQ({X_1_, X_2_, …, X_n_}) = <X_1_, X_2_, …, X_n_>.

Extending Chomsky’s (2021) analysis, we propose that, once the Subject coordination and the Predicate coordination are turned into n-tuples via FSQ, with conjuncts NP_1_, …, NP_n_ and V_1_, …, V_n_ discriminated according to their order, the correct S-P relations are assigned to each NP-V pair. That is, a special version of FS-P, call it FS-P′, is now applicable, conditioned by prior n-tuple formation via FSQ.

(10) FS-P′:
  In the configuration <NP_1_, …, NP_n_> … (Adverb_crossing_) … <V_1_, …, V_n_>, assign the S-P relation to <NP_i_, V_j_> (i.e., S-P<NP_i_, V_j_>), if i = j.

FS-P′ forms S-P<*Taro*, *sakebi*> and S-P<*Kenta*, *hanasiteiru*>, yielding the correct interpretation “Taro is shouting, and Kenta is uttering.”

Notice that FS-P (7) is rather directly applicable to Merge-based hierarchical structures {NP, V(P)} via Σ [Minimal Search, (6)]. This is not the case for FS-P′ (10), whose application for Crossing dependency is at best indirect and requires additional n-tuple formation (order assignment) via FSQ (9). As we will argue below, the differing complexity of these two types of computations can be experimentally elucidated by fMRI as responses in distinct regions of the brain.

### 1.2. Experimental design

**Figure 1:**
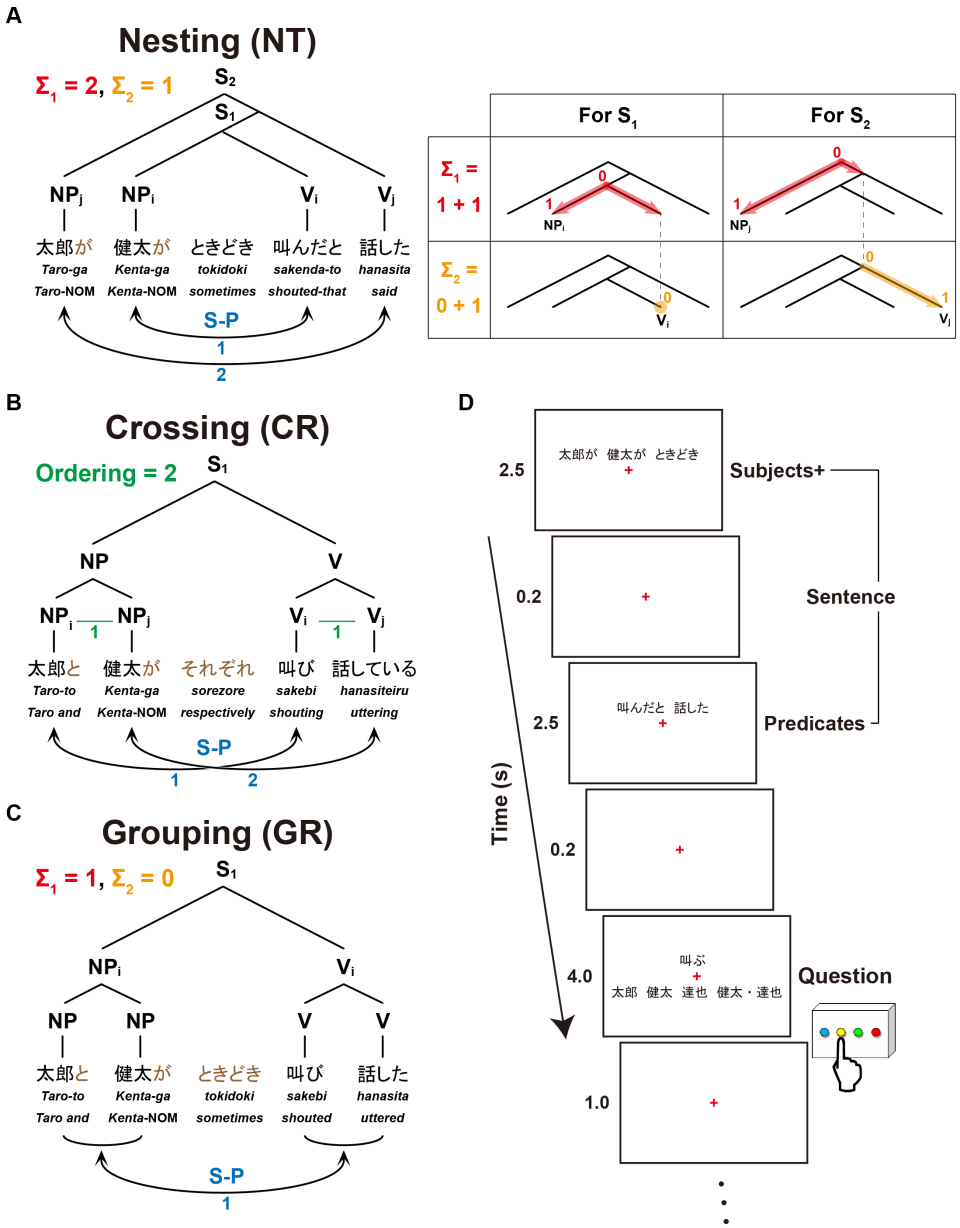
A paradigm for testing sentences with different dependencies. We tested three dependency types with two noun phrases (NPs) as Subjects and two verbs (Vs) as Predicates. Below each example sentence in Japanese, words in Romaji, each with translation in English, are shown. **(A)** Sentence structure with a Nesting (NT) dependency: “*Taro said that Kenta sometimes shouted*.” The linguistic operations of Σ_1_ (in red) and Σ_2_ (in orange) for the Nesting structure (see the Introduction for their definitions) are shown in the right panel. For S_1_, Σ_1_ searches the closest NP and identifies NP_i_ as the Subject; with NP_i_ fixed, Σ_2_ searches a V in the smallest set containing NP_i_ and identifies V_i_ as its Predicate. The same applies to S_2_. **(B)** Sentence structure with a Crossing (CR) dependency: “*Taro and Kenta are shouting and uttering* (e.g., on the phone)*, respectively*.” A horizontal line (in green) shown as “X–Y” (e.g., NP_i_–NP_j_, V_i_–V_j_) indicates that X and Y are linearly ordered; Ordering denotes the number of those sequential lines. **(C)** Sentence structure with a Grouping (GR) dependency: “*Taro and Kenta sometimes shouted and uttered* (e.g., in the park).” The same subscripts on NPs and Vs (e.g., NP_i_ and V_i_) indicate the Subject-Predicate (S-P) correspondence in the sentence (S), denoted by curved bidirectional arrows (numbers shown in blue). **(D)** An example of a single trial with stimulus events. A sentence was presented with NPs and an adverb (Subjects+) as the first half of stimuli, and with Vs (Predicates) as the latter half of stimuli. A question for asking about the S-P correspondence in that sentence followed. When a V was presented, the participants selected one of four choices including a group of NPs; when an NP was presented, they selected one of four choices including a group of Vs.

The present study focuses on three dependencies differing in complexity with respect to Σ_1_ (6a), Σ_2_ (6b), and FSQ (9). Nesting dependency (NT; Figure 1A) is the standard embedding configuration of Japanese, which forces center-embedding due to its canonical SOV order. In the NT configuration, Σ_1_ starts searching from the S_1_ node to pick an NP as the Subject; since S_1_ itself is not a possible option, Σ_1_ has to look deeper in the structure and pick up NP_i_ as a target [X in (6)]; being an NP, NP_i_ is identified as the Subject; then Σ_2_ applies to the sister node of NP_i_, picks the closest V/Predicate, namely V_i_, and assigns the relation S-P to the pair <NP_i_, V_i_> (i.e., S-P<NP_i_, V_i_>). Similarly, in the S_2_ cycle, Σ_1_ picks NP_j_, and then Σ_2_ applies to the set {S_1_, V_j_}, forming S-P<NP_j_, V_j_>. The digits on each node in the tree diagram indicate how many nodes Σ_1_ (in red) or Σ_2_ (in orange) passes before reaching that node from the top of the cycle. They thus indicate the *complexity* of the configuration in terms of Σ_1_ and Σ_2_: for the S_1_ cycle, Σ_1_ passes one node, and Σ_2_ does not pass any node because the sister node of NP_i_ itself is what needs to be selected; therefore, the complexity of S_1_ in terms of Σ_1_ is 1, and that in terms of Σ_2_ is 0. Along the same lines, the S_2_-cycle’s complexity in terms of Σ_1_ is 1, and the complexity in terms of Σ_2_ is 1. The total complexity of the configuration of Figure 1A is 1 + 1 = 2 for Σ_1_, and 0 + 1 = 1 for Σ_2_. The blue digits indicate how many times FS-P applies, here denoted by the factor of “S-P correspondence,” providing another measure of the sentence complexity.

Crossing dependency (CR; Figure 1B) was discussed above in (8). Since the standard type of Σ/FS-P cannot determine which conjunct is to be picked up by Σ, FSQ first applies to NPs and Vs, and then FS-P′ applies, forming the two S-P pairs: as the green lines and digits indicate, FSQ, denoted here by the factor of “Ordering,” determines ordering in each of the two pairs of elements, i.e., <NP_i_, NP_j_> and <V_i_, V_j_>; then FS-P′ forms S-P pairs <NP_i_, V_i_> and <NP_j_, V_j_>.

Grouping dependency (GR; Figure 1C) is the control condition. It is the simplest configuration of the three. It does not employ the Crossing-adverb *sorezore*. Thus, Σ_1_ can select {*Taro-to*, *Kenta-ga*} and Σ_2_ can pick {*sakebi*, *hanasita*}, forming S-P<{*Taro-to*, *Kenta-ga*}, {*sakebi*, *hanasita*}>. Σ/FS-P applies only once, yielding the interpretation “Taro and Kenta (sometimes) shouted and uttered.”

**Figure 2:**
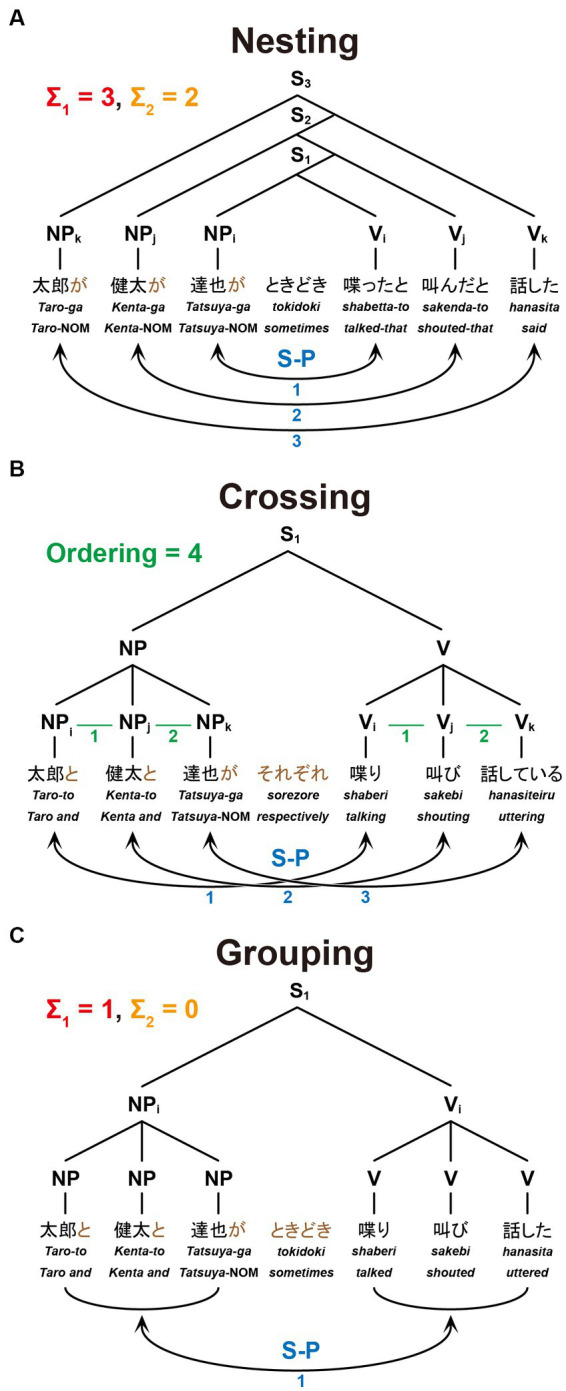
The structures and dependencies of six-word sentences. Nesting **(A)**, Crossing **(B)**, and Grouping **(C)** dependencies are shown in the same format as in Figure 1, together with example sentences: “*Taro said that Kenta shouted that Tatsuya sometimes talked*,” “*Taro, Kenta, and Tatsuya are talking, shouting, and uttering, respectively*,” and “*Taro, Kenta, and Tatsuya sometimes talked, shouted, and uttered*,” respectively.

In the experiments, we tested the recognition of dependencies for Japanese sentences by randomly presenting sentences with the above three dependency types to native speakers. It is assumed in theoretical linguistics that “in an NP-VP structure, NP and VP are generated in parallel, with no interaction” (Chomsky, 2021). A sentence was thus presented in two steps (Figure 1D): as stimulus events having NPs together with an adverb (denoted here as Subjects+), and as stimulus events having Vs (Predicates). We used four-word (4 W) and six-word (6 W) sentences (Figure 2) for each dependency type, which were also mixed. Only under the Nesting conditions, all NPs had a nominative case marker *-ga*, which indicates their status of Subject assigned by FS-P/Σ, whereas under the Crossing and Grouping conditions, the last NP alone had the particle -*ga* and the remaining NP(s) had a coordinator *-to* (“*and*”; see the brown letters in Figures 1, 2). Moreover, different sets of adverbs were used under the Crossing and Grouping conditions (see the Stimuli section). Therefore, Subjects+ provided sufficient information for predicting one of these six conditions, enabling structure building of a whole sentence (i.e., with Merge and Σ operations) in advance of the Predicates event. At the same time, the participants started to make possible S-P correspondences, thereby utilizing memorized Subjects+ and Predicates. During the presentation of a question, the participants were asked to select the corresponding NP(s) when a V was presented, whereas the participants selected the corresponding V(s) when an NP was presented. This novel paradigm was designed to reveal active and dynamic processes of structure building in terms of brain activations.

Estimates of linguistic and nonlinguistic factors to account for signal changes are summarized in Table 1. As explained above, the structure-based Σ_1_ and Σ_2_ operations apply during the Subjects+ events only under the Nesting and Grouping conditions, where their estimates under the Nesting conditions are greater than those under the Grouping conditions. Merge is theoretically “costless” (Saito and Fukui, 1998; Chomsky, 2004), and thus Merge itself was not regarded as a linguistic factor to account for signal changes in the present study. In contrast, Ordering processes occur during the Predicates events only under the Crossing conditions, when individual NPs presented during the Subjects+ events are recalled and paired with Vs in a specified linear order under the Crossing conditions (see Figure 1). S-P correspondence involves NP-V matching and retrieval of items from the memory stack; S-P correspondence is processed during the Subjects+ and Predicates events, unless there is no primary process other than basic S-P correspondence in a particular region. We regarded the Grouping conditions as the control for both behavior and activation analyses, and subtracted estimates under the Grouping conditions from those under the Nesting and Crossing conditions, separately for 6 W and 4 W conditions. We also considered nonlinguistic factors of memory span and numbers of encoding. Memory span is defined as the number of intervening words between a corresponding NP and V (e.g., four words excluding an adverb in-between NP_k_ and V_k_ in Figure 2A), which become fixed during the Predicates event, whereas numbers of encoded words were estimated separately for the Subjects+ and Predicates events. Subtracting these nonlinguistic factors under the Grouping conditions from those under the Nesting and Crossing conditions, the resultant estimates become zero or negative, and thus those factors were eliminated from our analyses.

**Table 1:** Estimates of various factors.

Linguistic factors												
	Subjects+						Predicates					
Factor	NT		CR		GR		NT		CR		GR	
	6 W	4 W	6 W	4 W	6 W	4 W	6 W	4 W	6 W	4 W	6 W	4 W
Σ_1_	3	2	–	–	1	1	–	–	–	–	–	–
Σ_2_	2	1	–	–	0	0	–	–	–	–	–	–
Σ_1_ + Σ_2_	5	3	–	–	1	1	–	–	–	–	–	–
Numbers of S-P	3	2	3	2	1	1	3	2	3	2	1	1
Numbers of Ordering	–	–	–	–	–	–	–	–	4	2	–	–
Degree of Merger	5	3	3	2	1	1	–	–	–	–	–	–
	NT – GR		CR – GR				NT – GR		CR – GR			
	6 W	4 W	6 W	4 W			6 W	4 W	6 W	4 W		
Σ_1_	2	1	–	–			–	–	–	–		
Σ_2_	2	1	–	–			–	–	–	–		
Σ_1_ + Σ_2_	4*	2*	–	–			–	–	–	–		
Numbers of S-P	2^†^	1^†^	2*^†^	1*^†^			2*^†^	1*^†^	2*	1*		
Numbers of Ordering	–	–	–	–			–	–	4^†^	2^†^		
Degree of Merger	4	2	2	1			–	–	–	–		
Nonlinguistic factors												
	Subjects+						Predicates					
Factor	NT		CR		GR		NT		CR		GR	
	6 W	4 W	6 W	4 W	6 W	4 W	6 W	4 W	6 W	4 W	6 W	4 W
Memory span	–	–	–	–	–	–	4	2	2	1	4	2
Numbers of encoding	3	2	3	2	3	2	3	2	3	2	3	2
	NT – GR		CR – GR				NT – GR		CR − GR			
	6 W	4 W	6 W	4 W			6 W	4 W	6 W	4 W		
Memory span	–	–	–	–			0	0	−2	−1		
Numbers of encoding	0	0	0	0			0	0	0	0		

Relative estimates for linguistic and nonlinguistic factors are shown separately for six-word (6 W) and four-word (4 W) sentences. For each factor, we define an individual estimate as the largest value that the factor can variably take within an entire sentence or a given domain. Among all tested conditions, the unit load for each factor should be invariable, making an independent subtraction between estimates of the same factor possible. Two estimates for 6 W and 4 W of the Grouping (GR) dependency were regarded as a reference and subtracted from their respective estimates of the Nesting (NT) or Crossing (CR) dependencies. An en-dash (−) denotes “not applicable,” which was regarded as null for the subtraction. Σ_1_ and Σ_2_ operations apply during the Subjects+ events only under the Nesting and Grouping conditions (see Figures 1, 2). S-P correspondence is processed during the Subjects+ and Predicates events, unless there is no primary process. Ordering processes occur during the Predicates events only under the Crossing conditions (see Figure 1B). The predicted estimates with asterisks (*) and daggers (†) were used to fit the activations in the bilateral LPMCs (Figures 5A,B) and L. FG/LG/MOG (Figure 5C), respectively. Regarding the concept of Degree of Merger, see Ohta et al. (2013). Memory span is defined as the number of intervening words between a corresponding NP and V. Numbers of encoding (i.e., encoded words) were estimated separately for the Subjects*+* and Predicates events.

Regarding the involvement of semantic factors, we carefully controlled them as much as we could in the experiment. What is generally called “the meaning/semantics of a sentence” is actually a result of interplay of two factors: (i) lexical semantics, i.e., what each lexical item refers to; (ii) hierarchical structures and formal relationships defined over them, such as Subject-Predicate correspondence. In the present experiment, all conditions use the same word-set except for the complementizer and coordinator (both realized as -*to* in Japanese), both of which are purely “functional” (“functional categories” in the technical terminology) in the sense that they do not contribute to semantics as lexical items. The factor (i) is thus controlled in all conditions. Also, the numbers of S-P correspondence were equivalent between Nesting and Crossing conditions (see Table 1), cancelling the effect (ii) except for the difference in formal structures. Hence, the only semantic difference between the three conditions is reduced to the structural difference, i.e., hierarchical structure and linear order formed by FSQ.

The most crucial regions for syntactic processing have been identified as the left lateral premotor cortex (L. LPMC) and left inferior frontal gyrus (L. IFG), which function as grammar centers (Sakai, 2005). The ventral L. IFG was shown to be crucially involved in the process of Merge-generable dependencies, whereas the L. LPMC and right (R.) LPMC were activated for Merge-generable and non-Merge-generable dependencies, both requiring S-P correspondence (Tanaka et al., 2019). The bilateral fusiform gyri (FG) and bilateral middle occipital gyri (MOG) were also activated for non-Merge-generable dependencies alone, where surface linear arrangements were artificially forced. The comparisons between the Nesting (totally Merge-generable) and Crossing (partially Merge-generable) dependencies in the current study would reveal further differences within Merge-generable dependencies among these regions. We hypothesize that the L. IFG and/or bilateral LPMCs primarily subserve Σ operations (required for the Nesting and Grouping conditions), and that the FG/MOG primarily subserve Ordering (required for the Crossing conditions alone). The bilateral lingual/fusiform gyri (LG/FG) have recently been discovered to be included in a network that regulates the higher-order cognitive functions (Tanaka et al., 2020), and that is independent from three (including the left LPMC, dorsal IFG, and ventral IFG) of the syntax-related networks (Kinno et al., 2014). We further hypothesize that all of the above regions can afford to process S-P correspondence if there are no such primary processes. Regarding the comparison between the Nesting and Crossing dependencies, the crucial distinction of cortical substrates would be elucidated for the core linguistic (i.e., structure-related) capacity and peripheral memory-based ordering, as well as for corresponding processes involving both linguistic and memory-based capacities.

## 2. Materials and methods

### 2.1. Participants

We recruited 32 native speakers of Japanese [11 females; age: 23.1 ± 6.2 years (mean ± standard deviation)], who showed right-handedness (laterality quotients: 81 ± 20) as determined by the Edinburgh inventory (Oldfield, 1971). None of the participants had neurological disorders. Prior to their participation in the study, written informed consent was obtained from each participant after the nature and possible consequences of the study were explained. Approval for the experiments was obtained from the ethical review committee for experimental research involving human subjects at Graduate School of Arts and Sciences, the University of Tokyo.

### 2.2. Stimuli and tasks

As visual stimuli, we prepared 30 grammatical and semantically natural sentences in Japanese for each of the following conditions: Nesting [six-words (6 W)], Nesting [four-words (4 W)], Crossing [6 W], Crossing [4 W], Grouping [6 W], and Grouping [4 W]. Every sentence with four words (excluding an adverb) had two noun phrases (NPs), an adverb, and two verbs (Vs) in the *surface order* of NP-NP-Adverb-V-V (see Figures 1A–C); those with six words were in the surface order of NP-NP-NP-Adverb-V-V-V (see Figure 2).

Each NP was presented using two letters of *kanji* (the adopted logographic Chinese characters used in written Japanese) that represent one of six common male names in Japanese (“*Taro*,” “*Jiro*,” “*Kenta*,” “*Shouta*,” “*Tatsuya*,” and “*Masaya*”). Each V was presented using one letter of *kanji* followed by two to four letters of *hiragana* (the basic Japanese syllabary that represents each mora in the Japanese language) according to its inflection. We used six verbs representing speech [“*hana-su*” (say/utter), “*shabe-ru*” (talk), “*sake-bu*” (shout), “*wame-ku*” (scream), “*tsubuya-ku*” (murmur), and “*sasaya-ku*” (whisper)], all of which can take a clausal complement in Japanese.

We used three types of grammatical particles, which represent canonical case markings and syntactic information in Japanese: the nominative case marker -*ga*, a coordinator -*to* (and), and a complementizer -*to* (that). Under the Nesting conditions, the complementizer was placed at the end of each V except the last one, and all Vs were presented in their past-tense form; the present-tense form was not used, because *-to* following a present tense verb is ambiguous, in that it sometimes means *if*/*then* as well as *that*. Under the Crossing and Grouping conditions, all Vs except the last one took their adverbial form, and each of them was followed by a Japanese punctuation mark in order to explicitly show conjunctives for the Vs. The last V under the Crossing conditions was presented in its progressive form in order to avoid group-reading (which collectively relates all NPs to all Vs as a group), since a single person cannot simultaneously perform the actions that each V indicates. In contrast, the last V under the Grouping conditions was presented in its past-tense form in order to ensure group-reading of a sequential act for all Vs.

Each adverb was presented using four letters of *hiragana*. For the adverbs under the Crossing conditions, we used different adverbs [“*sorezore*” (*respectively*), “*ono-ono*” (*every person*), and “*meimei*” (*individually*)], all of which function like *respectively* in English and naturally force Crossing dependency in a coordinate configuration. Although the Nesting and Grouping dependencies do not need a particular adverb, we used three adverbs representing frequency instead [“*tokidoki*” (*sometimes*), “*tabitabi*” (*repeatedly*), and “*shibashiba*” (*often*)]. The use of these words was randomized among the different sentences to alleviate any effects of particular adverbs.

In each trial, a whole sentence (“Sentence”) was serially presented in a pair of stimulus events, i.e., NPs together with an adverb (“Subjects+”) and Vs (“Predicates”) (see Figure 1D), preventing a direct visual linking of NPs and Vs. Stimuli during each of those events were visually presented with yellow characters for 3.5 s (6 W) or 2.5 s (4 W), with an interval of 0.2 s after each event. For the Subject-Predicate matching task, a question-set (“Question”) followed in one of two forms: (i) one V in the upper row and four NPs as options in the lower row; and (ii) one NP in the upper row and four Vs as options in the lower row. The NPs were presented without any particle, and the Vs were always in the present-tense form. One of the four options was a group of three (6 W) or two (4 W) words with middle dot(s), which was the correct answer to the Question for the Grouping conditions. The participants then selected one option within 6.0 s (6 W) or 4.0 s (4 W) by button pressing, judging the correct Subject-Predicate pair. Each trial lasted for 13.4 s (6 W) or 9.4 s (4 W) with a post-trial interval of 1.0 s.

The stimuli were presented against a dark background at the center of an eyeglass-like MRI-compatible display (resolution = 800 × 600 pixels, framerate = 60 fps; VisuaStim Digital, Resonance Technology Inc., Northridge, CA), and the participants wore earplugs. For fixation, a red cross was always shown at the center of the display, and the participants were instructed to keep their eyes on this position. The stimulus presentation and the collection of behavioral data [accuracy and response times (RTs)] were controlled using Presentation software (Neurobehavioral Systems, Albany, CA).

Participants were provided with an instruction sheet that included a sample sentence for each of the six conditions without showing its tree structure or S-P correspondence. The participants were trained outside the scanner with multiple sets of six trials (one trial per condition), until they correctly answered in at least five trials (per set) for two sets. During the MR scanning, no feedback on each trial’s performance was given to any participant. A single run of MR scans contained three trials per condition, i.e., 18 trials in total, whose order was randomized. For every participant, 14 runs were conducted with a brief break outside the scanner after the seventh run.

### 2.3. MRI data acquisition

For the MRI data acquisition, a participant was in a supine position, and his or her head was immobilized inside the radiofrequency coil. The MRI scans were conducted on a 3.0 T MRI system equipped with a bird-cage head coil (GE Signa HDxt 3.0 T; GE Healthcare, Milwaukee, WI). During the fMRI session, we scanned 30 axial 3-mm thick slices with a 0.5-mm gap, covering the volume range of −38.5 to 66 mm from the anterior to posterior commissure (AC-PC) line in the vertical direction, using a gradient-echo echo-planar imaging (EPI) sequence [repetition time (TR) = 2 s, echo time (TE) = 30 ms, flip angle (FA) = 78°, field of view (FOV) = 192 × 192 mm^2^, resolution = 3 × 3 mm^2^]. In a single scanning session, we obtained 119 volumes, and dropped the initial four volumes from analyses due to MR signal increases. High-resolution T1-weighted images of the whole brain (136 axial slices, 1 × 1 × 1 mm^3^) were acquired from all participants with a three-dimensional fast spoiled gradient-echo (3D FSPGR) acquisition in the steady state sequence (TR = 8.6 ms, TE = 2.6 ms, FA = 25°, FOV = 256 × 256 mm^2^). These structural images were used for normalizing the fMRI data.

### 2.4. fMRI data analyses

The fMRI data were analyzed in a standard manner using SPM12 statistical parametric mapping software^1^ (Wellcome Trust Center for Neuroimaging; Friston et al., 1995) implemented on MATLAB (Math Works, Natick, MA). The acquisition timing of each slice was corrected using the middle slice (the 15th slice chronologically) as a reference for the functional images. We spatially realigned each volume to the first volume of consecutive runs, and a mean volume was obtained. We set the threshold of head movement during a single run: within a displacement of 2 mm in any of the three directions, and within a rotation of 1.4° around any of the three axes. These thresholds were empirically determined from our previous studies (Kinno et al., 2008). If a run included one or several images over this threshold, we replaced the outlying image with an interpolated image, which was the average of the chronologically former and latter ones, and conducted the realignment procedure again. Data of five runs from the participants were excluded from analyses due to excessive head movement even after this procedure. The realigned data were resliced every 3 mm using seventh-degree B-spline interpolation. Each individual’s structural image was matched with the mean functional image generated during realignment. The resultant structural image was spatially normalized to the standard brain space as defined by the Montreal Neurological Institute (MNI) using the extended version of unified segmentation algorithm with light regularization; this is a generative model that combines tissue segmentation, bias correction, and spatial normalization in a single model (Ashburner and Friston, 2005). The resultant deformation field was applied to each realigned functional image in order to spatially normalize the images with non-linear transformation. All normalized functional images were then smoothed by using an isotropic Gaussian kernel of 9 mm full-width at half maximum (FWHM).

In a first-level analysis (i.e., the fixed-effects analysis) for each participant, hemodynamic responses were modeled with a boxcar function with a duration of 3.5 s (6 W) or 2.5 s (4 W) from the onset of each of the Subjects+ and Predicates events, as well as with a duration of 6 s (6 W) or 4 s (4 W) from the onset of Question. The boxcar function was then convolved with a hemodynamic response function, and low-frequency noises were removed by high-pass filtering at 1/128 Hz. To minimize the effects of head movement, the six realignment parameters obtained from preprocessing were included as a nuisance factor in a general linear model. The images for Subjects+, Predicates, and Question under each of six conditions were then generated in the general linear model for each participant, where activations under the Grouping conditions were further subtracted from those under the Nesting or Crossing conditions. Those subtracted images were then used for our intersubject comparison in a second-level analysis (i.e., the random-effects analysis).

In the second-level functional analyses with *t*-tests, we performed a two-way repeated measures analysis of covariance (rANCOVA) [dependency type (Nesting, Crossing) × word numbers (6 W, 4 W)] during Subjects+ or Predicates, as well as a three-way rANCOVA [dependency type × word numbers × stimulus event (Subjects+, Predicates)], with three nuisance factors (age, gender, and laterality quotient). The results were thresholded at uncorrected *p* < 0.001 for the voxel level, and at corrected *p* < 0.05 for the cluster level, with topological family-wise error (FWE) correction across the whole brain. For the anatomical identification of activated regions, we used the Anatomical Automatic Labeling method^2^ (Tzourio-Mazoyer et al., 2002) and the labeled data as provided by Neuromorphometrics Inc.,^3^ under academic subscription. In addition to whole-brain analyses, we adopted analyses of each region of interest (ROI) by using the MarsBaR-toolbox.^4^ We obtained ROI clusters from activations during the Subjects+ in the Nesting – Crossing contrast or Predicates in the Crossing – Nesting contrast. Regarding the cluster including the L. LPMC and supplementary motor area (SMA), we further extracted the L. LPMC with an AAL mask for the “Precentral” gyrus.

For activations in the ROIs, signal changes (averaged across participants) under the four conditions of Nesting [6 W], Nesting [4 W], Crossing [6 W], and Crossing [4 W] were fitted with the estimates for NT – GR [6 W], NT – GR [4 W], CR – GR [6 W], and CR – GR [4 W], respectively (see Table 1). We assumed a no-intercept model with a single scale parameter (*y* = *ax* + *b*; *b* = 0), and used a least-squares method to minimize the residual sum of squares (RSS) for the four fitted values (i.e., four estimates multiplied by the fitting scale, *a*) against corresponding signal changes. Goodness of fitting was further evaluated by using a one-sample *t*-test (significance level at *α* = 0.0125, Bonferroni corrected) between the fitted value for each contrast and individual activation.

## 3. Results

### 3.1. Behavioral data

**Figure 3:**
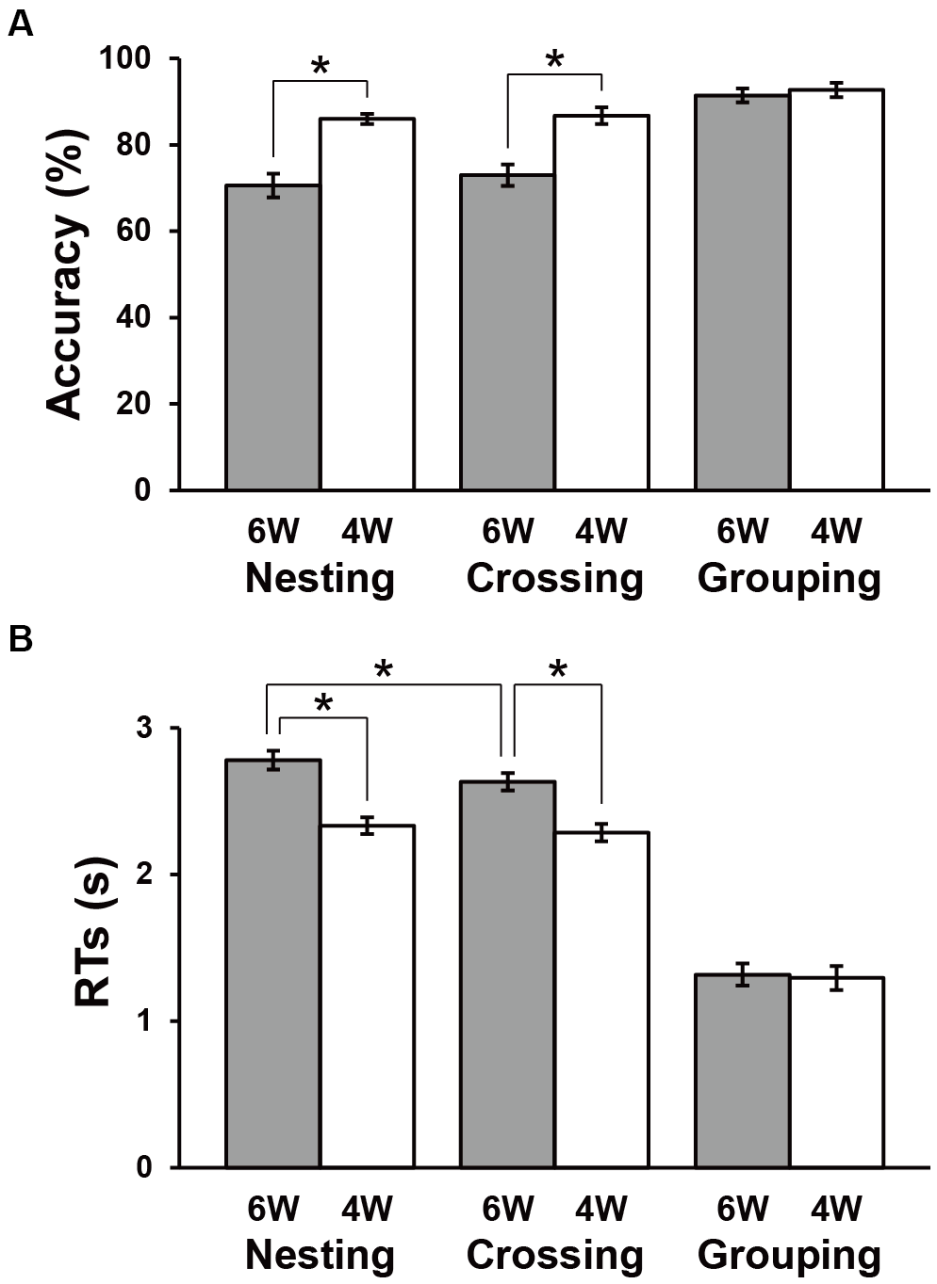
Behavioral data. Accuracies **(A)** and response times (RTs) **(B)** under each condition. Filled and open bars indicate data of six-word (6 W) and four-word (4 W) sentences, respectively. The Grouping conditions were the easiest, and thus used as a control in the below activation analyses. Error bars indicate standard errors of the mean (SEM). *p < 0.05.

The accuracy and RTs are shown in Figure 3. The Grouping conditions showed obviously higher accuracy and shorter RTs compared to the Nesting and Crossing conditions, consistent with the fact that the Grouping conditions were simplest. Paired *t*-tests showed no significant difference between 6 W and 4 W under the Grouping conditions (accuracy: *t*(31) = 0.9, *p* = 0.4; RTs: *t*(31) = 0.8, *p* = 0.4). Under the Nesting and Crossing conditions, a repeated measures analysis of variance (rANOVA) for the accuracy with two factors [dependency type (Nesting, Crossing) × word numbers (6 W, 4 W)] showed the significant main effect of word numbers [*F*(1, 31) = 63, *p* < 0.0001], but the main effect of dependency type [*F*(1, 31) = 1.2, *p* = 0.3] and the interaction between the two factors [*F*(2, 62) = 0.3, *p* = 0.6] were not significant (Figure 3A). Accuracies were significantly lower for 6 W than 4 W [Nesting: *t*(31) = 5.9, *p* < 0.0001; Crossing: *t*(31) = 6.9, *p* < 0.0001]. Regarding RTs, an rANOVA showed significant main effects of both dependency type [*F*(1, 31) = 15, *p* = 0.0006] and word numbers [*F*(1, 31) = 154, *p* < 0.0001], as well as a marginal interaction between them [*F*(1, 31) = 3.7, *p* = 0.06; Figure 3B]. Response times were consistently longer for 6 W than 4 W [Nesting: *t*(31) = 9.5, *p* < 0.0001; Crossing: *t*(31) = 10, *p* < 0.0001]. Moreover, RTs under the 6 W conditions were significantly longer for Nesting than Crossing [*t*(31) = 3.8, *p* = 0.0007]. These results indicate that the largest processing loads were associated with the Nesting 6 W condition, as expected. For subsequent activation analyses, we used both correct and incorrect trials, so that all participants and conditions tested were equally weighted in terms of the number of trials.

### 3.2. Differential cortical networks for processing Nesting and Crossing

**Figure 4:**
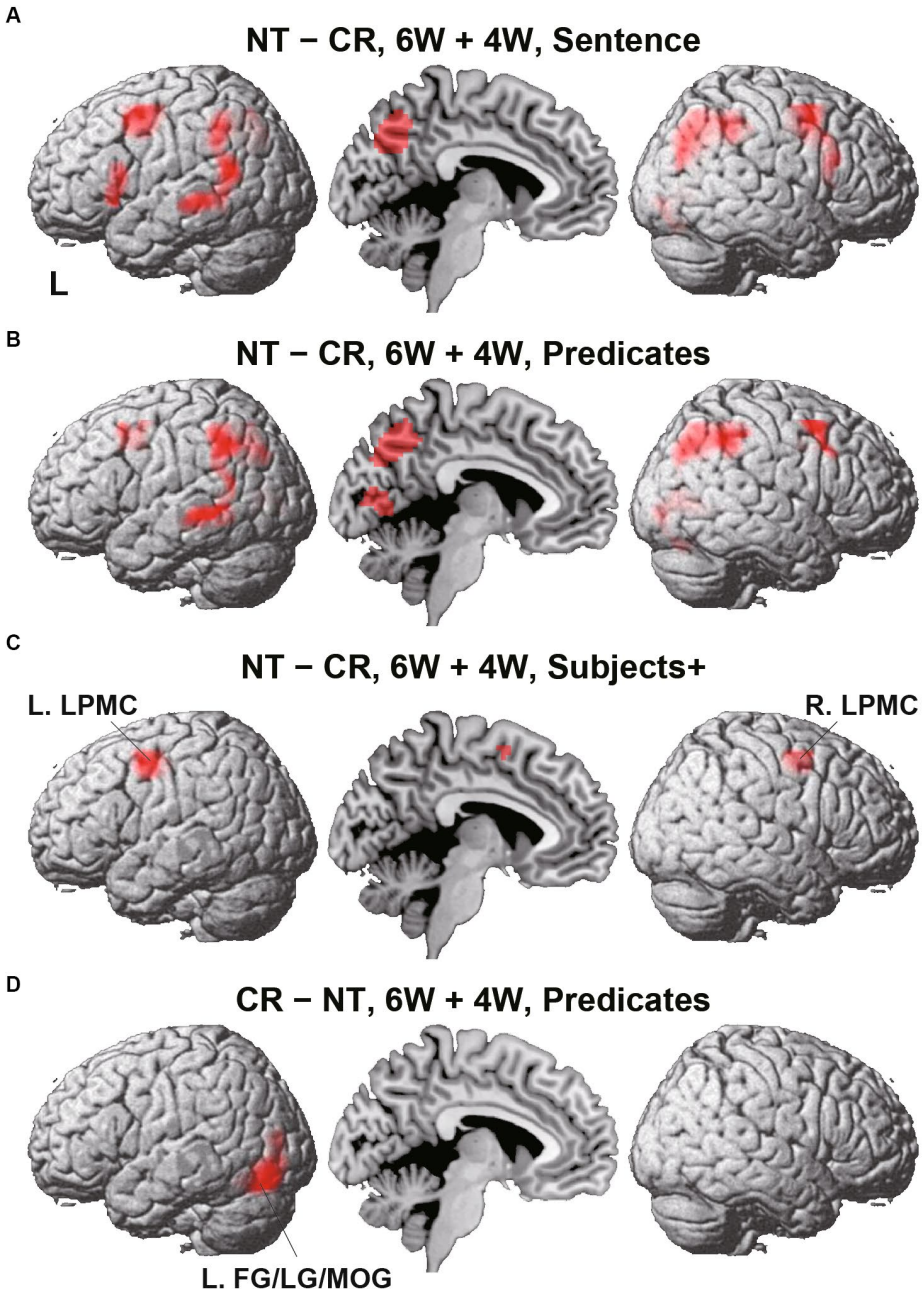
Direct comparisons between Nesting (NT) and Crossing (CR). **(A)** Results of the NT – CR contrast for the 6 W and 4 W sentences combined, during the presentation of the Sentence (combining Subjects+ and Predicates; see Figure 1D). Activations were shown in the left (L) lateral surface, mid-sagittal section, and right (R) lateral surface. **(B)** Results of the NT – CR contrast during Predicates. **(C)** Results of the NT – CR contrast during Subjects+. Activations were observed in the bilateral lateral premotor cortices (LPMCs). **(D)** Results of the reversed contrast of CR – NT during Predicates. Activations were observed in the left fusiform gyrus, lingual gyrus, and middle occipital gyrus (L. FG/LG/MOG). For each contrast, an exclusive mask (one-sample *t*-test, uncorrected *p* < 0.001) of negative activation for the CR (in **A-C**) or NT (in **D**) was applied. Significance was determined at uncorrected *p* < 0.001 for the voxel level, family-wise error (FWE) corrected *p* < 0.05 for the cluster level.

To identify cortical activations specific to the Nesting or Crossing conditions, we conducted direct comparisons between the two dependency types. Regarding the Nesting – Crossing contrast (6 W and 4 W combined) during the Sentence presentation (the stimulus event shown in Figure 1D), significant activations were observed in the bilateral LPMCs and IFGs, as well as in the left middle/inferior temporal gyri (MTG/ITG), bilateral angular/supramarginal gyri (AG/SMG), right MOG, right and medial precuneus, and right LG (Table 2; Figure 4A). These regions were major components of the syntax-related networks (Tanaka et al., 2020). We further separately analyzed activations during the Subjects+ and Predicates events. During the Predicates, an activation pattern similar to that during the Sentence was observed (Figure 4B). In addition, the medial LG and visual areas along the calcarine fissure (see the mid-sagittal section) were activated, presumably due to enhanced attention to the stimuli. During the Subjects+, activations were localized in the bilateral LPMCs and medial SMA (Figure 4C). In contrast, the reverse contrast of Crossing – Nesting resulted in focal activations in the L. FG, LG, and MOG during the Predicates (Figure 4D); these regions were activated during the Sentence as well, but not during the Subjects+. Hereinafter, we combine the L. FG/LG and MOG that consist the cluster of L. FG/LG/MOG. These results demonstrated the involvement of differential cortical networks for the Nesting and Crossing conditions.

**Table 2:** Regions with activations selective for the sentence presentation.

Brain regions	BA	Side	*x*	*y*	*z*	*Z*	voxel	*x*	*y*	*z*	*Z*	voxel
NT − CR, 6 W + 4 W			Sentence					Predicates				
LPMC	6/8	L	−39	2	44	5.7	443	−36	2	41	3.9	134
			−27	−4	53	5.6	*	−36	11	50	3.8	*
								−36	−4	56	3.5	*
IFG	44/45	R	54	20	20	3.8	553					
LPMC	6/8	R	27	2	50	6.3	*	33	8	56	4.4	186
								51	14	44	4.4	*
								33	−1	41	3.6	*
SFG/SMA	6/8	R						15	11	50	3.9	*
IFG	44/45	L	−60	14	14	4.1	141					
	45	L	−54	20	2	4.0	*					
	47	L	−36	20	−4	3.5	*					
MTG/ITG	21/20	L	−57	−31	−4	5.7	680	−63	−40	−4	5.1	588
			−57	−52	17	5.6	*	−45	−49	8	3.8	*
			−51	−43	−1	5.0	*	−60	−55	20	3.8	*
AG	39	L	−39	−46	44	4.9	*	−39	−52	53	4.0	*
SMG	40	L	−57	−43	32	3.8	*	−48	−52	38	5.2	*
MOG	18/19	R	39	−76	29	5.4	1,367					
			18	−67	23	4.4	*	12	−70	26	3.3	1,538
AG	39	R	33	−46	38	4.7	*	39	−58	50	4.4	*
SMG	40	R	60	−34	44	3.3	*	48	−49	50	5.6	*
Precuneus	7	R	15	−73	47	7.1	*	15	−70	53	6.5	*
		M	−9	−70	44	5.6	*	−9	−67	53	4.6	*
								−6	−70	41	4.6	*
								−9	−55	44	4.1	*
Calcarine	17/18	M						12	−76	5	6.9	*
								−6	−82	8	3.8	*
LG	18/19	R	15	−85	−7	6.6	205	15	−85	−4	7.3	*
			15	−73	2	4.3	*	27	−55	−1	4.3	*
		M						−6	−70	5	4.8	*
Cerebellum VI		R						15	−79	−19	4.8	*
Vermis		M						6	−70	−16	3.3	*
NT − CR, 6 W + 4 W			Subjects+									
LPMC	6/8	L	−21	−4	53	5.2	288					
			−39	−1	47	4.4	*					
SMA	6	M	−6	2	59	3.5	*					
LPMC	6/8	R	27	−1	56	5.5	153					
CR − NT, 6 W + 4 W			Predicates									
FG	37	L	−30	−70	−13	5.6	483					
LG	18/19	L	−18	−79	−10	5.2	*					
MOG	18/19	L	−27	−82	11	4.9	*					
			−30	−88	−1	3.6	*					

Stereotactic coordinates (*x*, *y*, *z*) in the MNI space are shown for activation peaks of *Z* values, which were more than 12 mm apart (see Figure 4). The region with an asterisk is included within the same cluster shown in the nearest row above. BA: Brodmann’s area; L: left; R: right; M: medial; AG: angular gyrus; FG: fusiform gyrus; IFG: inferior frontal gyrus; LG: lingual gyrus; LPMC: lateral premotor cortex; MOG: middle occipital gyrus; MTG/ITG: middle/inferior temporal gyri; SFG: superior frontal gyrus; SMA: supplementary motor area; SMG: supramarginal gyrus.

**Figure 5:**
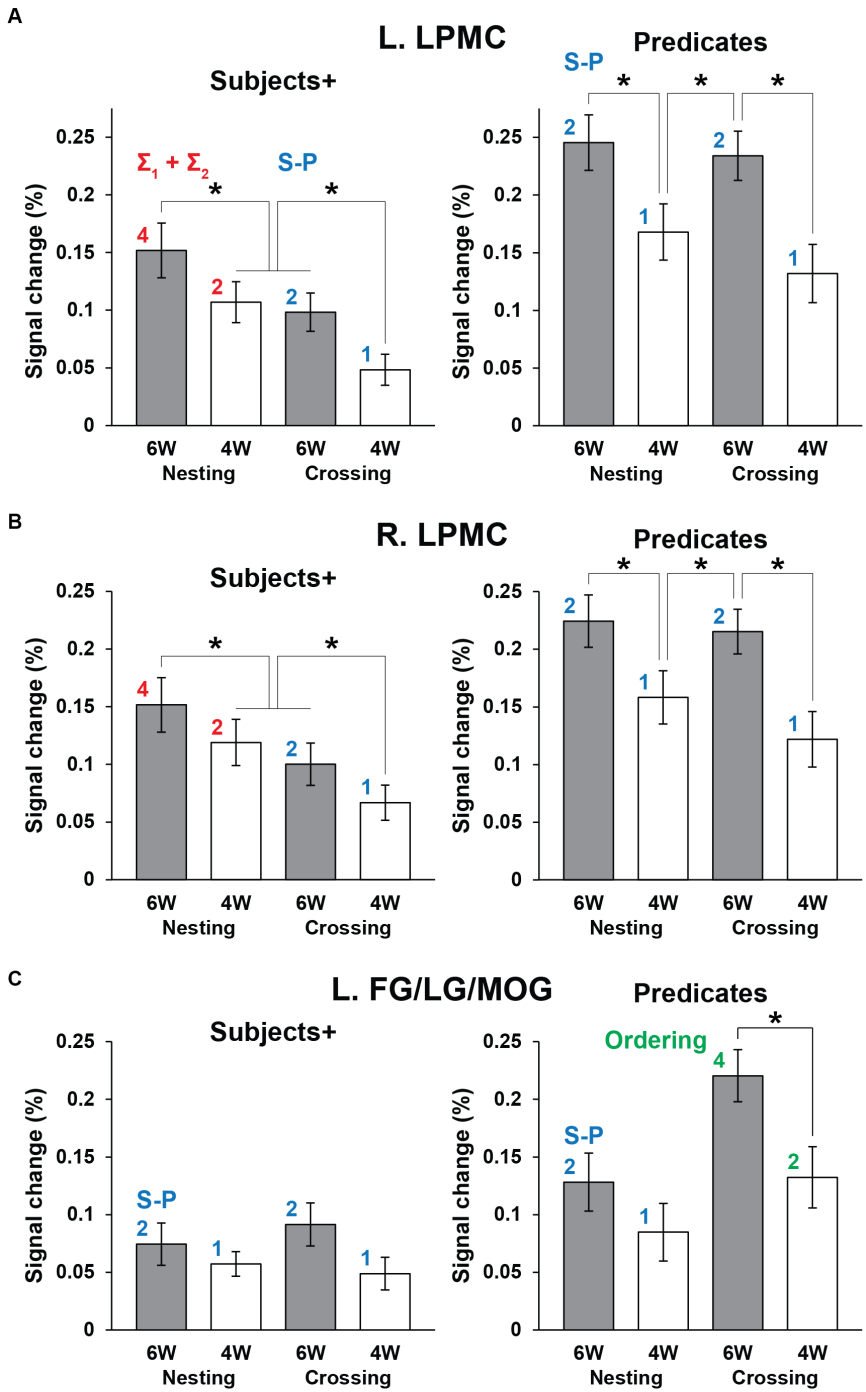
The distinctive activation patterns between the Subjects+ and Predicates. **(A)** Activations in the L. LPMC under each condition. For each panel, the red and blue digits/labels denote the estimates for fitting (asterisks in Table 1). **(B)** Activations in the R. LPMC, which were similar to those in the L. LPMC. **(C)** Activations in the L. FG/LG/MOG. For each panel, the blue and green digits/labels denote the estimates for fitting (daggers in Table 1). For all regions, activations for the Grouping conditions as a baseline were subtracted from those for the Nesting and Crossing conditions. Error bars indicate SEM. *p < 0.05.

Next we focused on the cortical regions, which were observed during the Sentence as well as either Subjects+ or Predicates, ensuring robustness and consistency among activated regions. In the Nesting – Crossing contrast (see Figures 4A–C), we defined two ROIs of L. LPMC and R. LPMC based on the extent of activations shown in Figure 4C. Regarding the Grouping conditions as a baseline, signal changes were calculated separately under the 6 W and 4 W conditions (Figures 5A,B). For the L. LPMC during the Subjects+, an rANOVA with two factors showed significant main effects of both dependency type [*F*(1, 31) = 15, *p* = 0.0005] and word numbers [*F*(1, 31) = 6.0, *p* = 0.02], with no significant interaction between them [*F*(1, 31) = 0.06, *p* = 0.8]. The signal changes were comparable between Nesting [4 W] and Crossing [6 W]; averaged signal changes under these two conditions were significantly lower than those under Nesting [6 W] [*t*(31) = 2.7, *p* = 0.01], and higher than those under Crossing [4 W] [*t*(31) = 3.2, *p* = 0.003]. Regarding the Predicates, the L. LPMC activations were enhanced compared to those during the Subjects+, especially under the 6 W conditions (*p* < 0.01). An rANOVA with two factors showed significant main effects of both dependency type [*F*(1, 31) = 12, *p* = 0.002] and word numbers [*F*(1, 31) = 24, *p* < 0.0001], with no significant interaction between them [*F*(1, 31) = 1.9, *p* = 0.2]. Paired *t*-tests confirmed that the signal changes were greater under the 6 W than under the 4 W conditions (Nesting [6 W] vs. Nesting [4 W]: *t*(31) = 3.8, *p* = 0.0007; Crossing [6 W] vs. Nesting [4 W]: *t*(31) = 3.5, *p* = 0.001; Crossing [6 W] vs. Crossing [4 W]: *t*(31) = 5.1, *p* < 0.0001). All of these results regarding the L. LPMC were statistically replicated for the R. LPMC (Figure 5B).

We also conducted ROI analyses on the L. FG/LG/MOG (Figure 5C), based on the extent of activations for the Crossing – Nesting contrast (see Figure 4D). During the Subjects+, an rANOVA showed significant main effects of neither dependency type [*F*(1, 31) = 0.2, *p* = 0.7] nor word numbers [*F*(1, 31) = 2.8, *p* = 0.1], and no significant interaction between them [*F*(1, 31) = 0.7, *p* = 0.4]. Regarding the Predicates, an rANOVA showed significant main effects of both dependency type [*F*(1, 31) = 46, *p* < 0.0001] and word numbers [*F*(1, 31) = 8.9, *p* = 0.006], with no significant interaction between them [*F*(1, 31) = 3.3, *p* = 0.08]. Paired *t*-tests confirmed that signal changes under the Crossing [6 W] condition were larger than those under the Crossing [4 W] condition [*t*(31) = 3.3, *p* = 0.003], and also larger than those under Nesting [6 W] and Nesting [4 W] (*p* < 0.0001).

We tested the models proposed in the Introduction, such that the bilateral LPMCs primarily subserve Σ operations, and that the FG/LG/MOG primarily subserve Ordering. We also assumed that these regions process S-P correspondence if there are no such primary processes; we thus regarded “numbers of S-P” as secondary factors. Regarding the L. LPMC and R. LPMC for the Subjects+, we combined “Σ_1_ + Σ_2_” for the Nesting conditions (i.e., 4: 2) with “numbers of S-P” for the Crossing conditions (i.e., 2: 1), resulting in the estimates of “4: 2: 2: 1.” Similarly, for the Predicates when there was no primary process, the estimates of “numbers of S-P” were used for all conditions (i.e., 2: 1: 2: 1). These models well explained the signal changes, exhibiting low values of RSS (≤ 0.002) and high coefficients of determination (*r^2^* ≥ 0.94; Table 3).

**Table 3:** Likelihood of fitting activations to each estimate.

Region	Stimulus event	Estimates	RSS	*r* ^2^	*p*-values for fitting	Log-likelihood	Likelihood ratio
L. LPMC	Subjects+	4: 2: 2: 1	0.0010	0.97	0.23, 0.43, 0.44, 0.67	25.0	1.0
		2: 1: 2: 1	0.0035	0.90	0.026, 0.058, 0.21, 0.39	19.3	3.4 × 10^−3^
	Predicates	2: 1: 2: 1	0.0022	0.98	0.094, 0.41, 0.80, 0.81	21.6	1.0
		2: 1: 4: 2	0.022	0.81	0.0004*, 0.0006*, 0.0058*, 0.51	10.8	2.1 × 10^−5^
R. LPMC	Subjects+	4: 2: 2: 1	0.0022	0.94	0.14, 0.15, 0.28, 0.55	21.5	1.0
		2: 1: 2: 1	0.0041	0.89	0.018, 0.049, 0.56, 0.88	18.6	5.7 × 10^−2^
	Predicates	2: 1: 2: 1	0.0022	0.98	0.077, 0.40, 0.74, 0.81	21.6	1.0
		2: 1: 4: 2	0.019	0.81	0.0005*, 0.0006*, 0.0049*, 0.53	11.5	4.0 × 10^−5^
L. FG/LG/MOG	Subjects+	2: 1: 2: 1	0.0004	0.97	0.21, 0.48, 0.72, 0.84	29.4	1.0
		4: 2: 2: 1	0.0030	0.80	0.041, 0.11, 0.13, 0.59	20.1	9.1 × 10^−5^
	Predicates	2: 1: 4: 2	0.0012	0.98	0.32, 0.44, 0.62, 0.72	24.2	1.0
		2: 1: 2: 1	0.0061	0.91	0.037, 0.11, 0.13, 0.79	16.8	5.8 × 10^−4^

Percent signal changes in each region (L. LPMC, R. LPMC, and L. FG/LG/MOG) were fitted with a single scale parameter to a model of each factor using its subtracted estimates (see Table 1; Figure 5) for the four contrasts of NT [6 W], NT [4 W], CR [6 W], and CR [4 W]. A least-squares method (coefficient of determination: *r*^2^) was used to minimize the residual sum of squares (RSS) for the four fitted values against corresponding signal changes. The *p*-values for the one-sample *t*-tests are shown in ascending order. For references in each region, we switched from the good model to an alternative model by exchanging the estimates between bilateral LPMCs and L. FG/LG/MOG. For each region, a likelihood ratio is shown as the ratio of each model’s likelihood to that of the good model during Subjects+ or Predicates.

* *p* < 0.0125 (Bonferroni corrected).

Regarding the L. FG/LG/MOG for the Predicates, we combined Ordering for the Crossing conditions (i.e., 4: 2) with “numbers of S-P” for the Nesting conditions (i.e., 2: 1), resulting in the estimates of “2: 1: 4: 2” (in the order of Nesting and Crossing). Similarly, for the Subjects+ when there was no primary process, the estimates of “numbers of S-P” were used for all conditions (i.e., 2: 1: 2: 1). These models also explained the signal changes, exhibiting low values of RSS (≤ 0.001) and high coefficients of determination (*r^2^* ≥ 0.97). In the bilateral LPMCs and L. FG/LG/MOG, the *p*-values were always larger than 0.07, without significant differences.

For references in each region, we switched from the above-mentioned good model to an alternative model, by exchanging the estimates between bilateral LPMCs and L. FG/LG/MOG (e.g., for Subjects+, 2: 1: 2: 1 in the L. LPMC, and 4: 2: 2: 1 in the L. FG/LG/MOG). For the alternative models, the *p*-values on Predicates were significantly smaller in the bilateral LPMCs, indicating that the alternative models were unlikely. By calculating a likelihood ratio as the ratio of the alternative model’s likelihood to the good model’s likelihood (log-likelihood is shown in Table 3), we found that the alternative models were by far less likely than the good ones (≤ 5.7 × 10^−2^). Therefore, the bilateral LPMCs and L. FG/LG/MOG showed a *double dissociation* for primary processes with Σ (under the Nesting conditions) and Ordering (under the Crossing conditions), respectively.

## 4. Discussion

By employing a novel paradigm to manipulate dependencies occurring in a sentence (Figures 1, 2), we obtained the following three striking results. First, for the Nesting – Crossing contrast, significant activations were observed in the bilateral LPMC and IFG, as well as in the left middle temporal gyrus and bilateral angular/supramarginal gyri (Figure 4), indicating engagement of the syntax-related networks. In contrast, the Crossing – Nesting contrast showed focal activations in the L. FG/LG/MOG. Secondly, during the Subjects+ events under the Nesting conditions, signal changes in bilateral LPMCs were well fitted with the estimates of computational costs (Σ_1_ + Σ_2_) to search the workspace and to select items (Table 3; Figure 5). Moreover, during the Predicates events under the Crossing conditions, the signal changes in the L. FG/LG/MOG were differentially fitted with the estimates of loads related to the ordering of elements/words (numbers of Ordering). Thirdly, these fitting models were by far more likely than the exchanged estimates between bilateral LPMCs and L. FG/LG/MOG (Table 3), confirming a double dissociation for primary processes with Σ (under the Nesting conditions) and Ordering (under the Crossing conditions). In conclusion, these results indicate that separate cortical networks are differentially employed, and their careful elucidation will provide further insights and challenges.

We have previously proposed that the structural depth of a sentence could be represented as the Degree of Merger (DoM) to account for the L. IFG activations (Ohta et al., 2013). As shown in Table 1, the factor of DoM also resulted in the estimates of “4: 2: 2: 1” during Subjects+ (for NT – GR and CR – GR), modeling the bilateral LPMC activations in a manner consistent with the accounting provided by Σ and numbers of S-P (Figures 5A,B). In other words, we expanded the DoM model slightly, and incorporated it into the theoretical foundation with Σ and FS-P on the basis of tree structures of a sentence. Given that adjacent regions in the association cortex receive inputs from multiple sources (Romanski, 2004), which are further integrated into individual regions, we hypothesize that those regions subserve at least double functions. In our previous study (Ohta et al., 2013), we combined two factors of DoM and numbers of S-P (“Search” in that paper) to account for the L. SMG activations. In the present study, when the bilateral LPMCs are not engaged in Σ operations under the Crossing conditions during the Subjects+ (see Table 1), these regions process information of S-P correspondence, just like during the following Predicates (Figures 5A,B). Similarly, when the L. FG/LG/MOG are not engaged in Ordering processes under the Nesting conditions during Predicates, these regions take over the process of S-P correspondence (Figure 5C). The double functions of these regions are reminiscent of the “filling-in” mechanisms among adjacent cortical regions (Gerrits and Vendrik, 1970; Komatsu, 2006), in which the regions having anatomical connections with the “silent” area (the blind spot or lesion) of the visual field, i.e., those not engaged in primary processes, take over the processes of peripheral regions with different inputs.

In our previous study, which tested S-P correspondence in a sentence with various structural depths, selective activations were observed in the L. IFG and L. SMG for Nesting sentences compared to simple sentences (Ohta et al., 2013). Moreover, the R. LPMC was activated by the direct contrast of reverse order vs. same (non-reverse) order for the sequence of pseudowords or letter strings, irrespective of the presence of hierarchical/recursive structures (cf. Figures 5C,D in Ohta et al., 2013), which could be explained by memory span as well as the executive control loads that become larger for reverse order. This contrast is similar to Nesting – Crossing in the present study, and we showed another possibility that the bilateral LPMC activations were explained by S-P correspondence. As mentioned in the Introduction, the bilateral LPMCs were activated consistently for Merge-generable and non-Merge-generable dependencies, both requiring S-P correspondence (Tanaka et al., 2019). In addition, the prefrontal cortex has been implicated in *sequential* processing, as modeled by non-human primates executing arm movements assigned to individual cursor movements in a path-planning task, as well as executing complex motor sequences based on memory (Tanji et al., 2007). Such animal studies suggest that the prefrontal cortex plays general roles in establishing correspondence between visual cues and sequences, similar to the S-P correspondence subserved by the bilateral LPMCs.

The FG subserves several functions, best known for its role in the discrimination of visually presented word forms (McCandliss et al., 2003; Dehaene and Cohen, 2011; Hirshorn et al., 2016), as well as of faces and objects (Kanwisher et al., 1997; Gauthier et al., 1999; Dickerson et al., 2007; Kale et al., 2019). A possible general role of the FG would be to distinguish similar elements of the same category, which was crucial for the ordering of grouped elements under the Crossing conditions in the present study. A previous fMRI study reported activations in the R. FG/LG/MOG for remembering an order of visual spots presented to either left or right eye (Rosenthal et al., 2016), and we have previously reported bilateral activations in the FG/MOG for matching the sequence of NPs and that of Vs (Tanaka et al., 2019). In the present study, we observed differential activations, such that L. FG/LG/MOG was activated under the Crossing conditions, while R. LG/MOG was activated under the Nesting conditions (see Table 2). Further studies are needed to clarify such a lateralization of this region in the human brain.

The left-lateralized regions of LPMC, dorsal IFG (the opercular/triangular parts of the F3; F3op/F3t), ventral IFG (the orbital part of the F3; F3O), MTG/ITG, and AG/SMG are known to be critically involved in linguistic functions (Sakai, 2005). We observed left-lateralized MTG/ITG activations for the Nesting – Crossing contrast, while activations in the IFG, LPMC, and AG/SMG were bilateral. All of these regions constitute the syntax-related networks identified by using a picture-sentence matching task (Kinno et al., 2014; Tanaka et al., 2020). While the bilateral regions of the dorsal IFG belong to Network I (one of syntax-related networks), the R. LPMC and L. LPMC belong to Networks I and II, respectively, each of which is anatomically and functionally connected. It is interesting to note that the bilateral LPMCs showed the same patterns of activations (see Figure 5), although their functional roles are different in the networks. The IFG regions were also bilaterally activated during the Sentence event (see Figure 4A), suggesting processes of integrating NPs and Vs into syntactic structures. These integration processes are better highlighted by sequential presentation of individual words, as shown by our previous experiment (Ohta et al., 2013), in which DoM actually explained the left-lateralized IFG activations. Moreover, the left AG has been implicated in semantic processes particularly at the sentence level (Humphries et al., 2007; Lau et al., 2008). The interactions among these language-related regions in both hemispheres, and how specific linguistic information is exchanged among them, should be further examined in the future study.

The present results strongly suggest that specific regions (L. FG/LG/MOG)—distinct from the one which processes Merge (L. IFG; see Sakai (2005) and Tanaka et al. (2019), among others)—are employed to compute the representations yielded by FSQ. As explained in the Introduction section, Nesting, Grouping, and Crossing dependencies are all Merge-generable, in the sense that they are determined over the structures built by Merge: the application of FS-P to the hierarchical structure generated by Merge directly derives Nesting and Grouping dependencies; Crossing dependency, on the other hand, additionally requires FSQ, in order for FS-P′ to distinguish NP-/V-conjuncts to form correct S-P pairs. The crucial difference between the Nesting and Crossing dependencies lies in the application of FSQ, for which direct comparisons of Crossing – Nesting revealed substrates of the L. FG/LG/MOG.

Given the distinct neural bases for processing Merge and FSQ, we gain a completely different perspective on the characterization of the human language faculty than the one that has been widely assumed in the literature. Natural language syntax has long been characterized in terms of the Chomsky Hierarchy (Chomsky, 1959, 1963; Fukui, 2015; Tanaka et al., 2019). It has been demonstrated that context-free phrase structure grammar (CFPSG) can generate almost all strings observed in natural language, including Nesting dependency (as exemplified by “mirror-image language,” e.g., *abba*, *aabbaa, abbbba, …*; Chomsky, 1957). However, there is an exception: Crossing dependency (cf. Chomsky’s “copying language,” e.g., *abab, aabaab, abbabb, …*) falls outside the class determined by CFPSG; it requires a “more powerful” type of rules. Chomsky (1957, 1959) argues that grammatical transformations, which are amply justified on independent grounds as a necessary descriptive device for human language, successfully handle Crossing dependency as well. Most linguists and computer scientists, however, have tended to avoid having recourse to transformational grammar (which they generally regard as mathematically ill-understood), and have claimed instead that context-sensitive phrase structure grammar (CSPSG), which is “one-step higher” than CFPSG in the Chomsky Hierarchy, is necessary for treating Crossing dependency. As is well-known, while Nesting dependencies abound in human language, Crossing dependencies are rather rare, occurring only in very limited constructions. Thus, syntacticians have considered human language to be “mildly” context-sensitive. According to this view, human language syntax is located “somewhere between” CFPSG and CSPSG—not a very satisfactory situation.

We can now offer a much clearer view on this issue. Quite apart from the Chomsky Hierarchy, as we briefly discussed in the Introduction in regard to Merge-generability, essentially all the core syntactic properties of human language can be captured by the simple structure-building operation Merge (which embodies the relevant characteristics of phrase structure rules and transformations postulated in earlier frameworks). However, a tiny portion of human language constructions are of an exceptional character that seems to require a reference to the linear order of elements, and FSQ is employed in these rare cases. Crossing dependency is one of these exceptional constructions. Thus, the traditional divide between CFPSG and CSPSG, along with their relative standing in the Chomsky Hierarchy, is illusory and hence beside the point, as far as human language is concerned. The existence of a small number of Crossing dependencies in a limited domain of syntax does not indicate the need for CSPSG, but rather, represents an exceptional fragment of constructions in human language in which linear order plays a role. For further discussion on this and related topics, see Fukui (2015) and Tanaka et al. (2019).

As demonstrated in detail in Tanaka et al. (2019), the Merge-generability Hypothesis elegantly characterizes human language. Recall that Nesting and Crossing dependencies are both Merge-generable: the two dependencies are defined over the structures Merge generates, but with the crucial aid of FSQ for Crossing dependency, since FS-P′, the relevant interpretive rule for Crossing dependency, requires linear/sequential matching [cf. (10)]. This is the fact that Tanaka et al. experimentally established, but they were not able to find a region responsible for the calculation of FSQ-generated structures. The present study specifies where the relevant regions are located, and thus confirms the Merge-generability Hypothesis, which predicts that Crossing dependency is formed by Merge and FSQ.

Crossing dependency, in our view, is a consequence of the fact that a somewhat “elementary” (presumably “pre-linguistic” in human evolutionary history; cf. the discussion above on the general role of L. FG) order-forming capacity permeates into the “peripheral” domain of syntax (see Chomsky, 2021). As we discussed in the Introduction with reference to the structure-dependence of the agreement rule in English, linear order is not the governing factor for core operations of human language. Rather, FSQ—and the interpretive rule based on FSQ (i.e., FS-P′)—only supplements the Merge-based core syntax, applying to the hierarchical structures Merge generates, in order to help obtain a specific, and rather peculiar, type of interpretations. It is thus important to emphasize once again that what matters most in human language is structure, not linear order. In fact, looking at the situation regarding Nesting and Crossing dependencies from a slightly different angle, one might argue that the rarity of Crossing dependency in human language, as opposed to the quite regular and frequent occurrences of Nesting dependency, indicates that unlike the latter, the former type of dependency—and consequently, the concept of linear order that it necessarily involves—does not directly reflect the core property of human language. If it did, the fact that its distribution is heavily restricted in highly specific constructions would be left a mystery.

## 5. Conclusion

To sum up, the central result of our present experimental study not only confirms the Merge-generability Hypothesis of Tanaka et al. (2019), but essentially completes the overall picture provided by the Hypothesis regarding the nature of syntactic dependencies in human language. Differential cortical networks have now been identified for the core structure-related capacity and peripheral memory-based ordering. Further challenges remain in elucidating how the two separate systems interact, and which sources are provided for linguistic and memory-based processes that support human intellectual capacities.

## Data availability statement

The raw data supporting the conclusions of this article will be made available by the authors, without undue reservation.

## Ethics statement

The studies involving human participants were reviewed and approved by the ethical review committee for experimental research involving human subjects at Graduate School of Arts and Sciences, the University of Tokyo. The participants provided their written informed consent to participate in this study.

## Author contributions

IN and KU prepared the linguistic stimuli. KU conducted the experiments. KU and KLS analyzed the data. All authors contributed to the article and approved the submitted version.

## Funding

This research was supported by Grants-in-Aid for Scientific Research (B) (Nos. JP17H02347, JP19H01256, and JP21H00532), a Grant-in-Aid for JSPS Fellows (No. JP22J21631), and Grants-in-Aid for Challenging Research (Pioneering No. JP21K18115 and Exploratory No. 21K18367) from the Ministry of Education, Culture, Sports, Science, and Technology of Japan.

## Conflict of interest

The authors declare that the research was conducted in the absence of any commercial or financial relationships that could be construed as a potential conflict of interest.

## Publisher’s note

All claims expressed in this article are solely those of the authors and do not necessarily represent those of their affiliated organizations, or those of the publisher, the editors and the reviewers. Any product that may be evaluated in this article, or claim that may be made by its manufacturer, is not guaranteed or endorsed by the publisher.

## Footnotes

^1^ http://www.fil.ion.ucl.ac.uk/spm/

^2^ http://www.gin.cnrs.fr/AAL2/

^3^ http://Neuromorphometrics.com/

^4^ http://marsbar.sourceforge.net/
